# A Computed Tomography Nomogram for Assessing the Malignancy Risk of Focal Liver Lesions in Patients With Cirrhosis: A Preliminary Study

**DOI:** 10.3389/fonc.2021.681489

**Published:** 2022-01-21

**Authors:** Hongzhen Wu, Zihua Wang, Yingying Liang, Caihong Tan, Xinhua Wei, Wanli Zhang, Ruimeng Yang, Lei Mo, Xinqing Jiang

**Affiliations:** ^1^ Department of Radiology, The First Affiliated Hospital of Jinan University, Guangzhou, China; ^2^ Department of Radiology, Guangzhou First People’s Hospital, School of Medicine, South China University of Technology, Guangzhou, China; ^3^ Department of Radiology, Foshan Hospital of Traditional Chinese Medicine, Foshan, China

**Keywords:** focal liver lesion, liver, computed tomography, nomogram, neoplasm

## Abstract

**Purpose:**

The detection and characterization of focal liver lesions (FLLs) in patients with cirrhosis is challenging. Accurate information about FLLs is key to their management, which can range from conservative methods to surgical excision. We sought to develop a nomogram that incorporates clinical risk factors, blood indicators, and enhanced computed tomography (CT) imaging findings to predict the nature of FLLs in cirrhotic livers.

**Method:**

A total of 348 surgically confirmed FLLs were included. CT findings and clinical data were assessed. All factors with P < 0.05 in univariate analysis were included in multivariate analysis. ROC analysis was performed, and a nomogram was constructed based on the multivariate logistic regression analysis results.

**Results:**

The FLLs were either benign (n = 79) or malignant (n = 269). Logistic regression evaluated independent factors that positively affected malignancy. AFP (OR = 10.547), arterial phase hyperenhancement (APHE) (OR = 740.876), washout (OR = 0.028), satellite lesions (OR = 15.164), ascites (OR = 156.241), and nodule-in-nodule architecture (OR =27.401) were independent predictors of malignancy. The combined predictors had excellent performance in differentiating benign and malignant lesions, with an AUC of 0.959, a sensitivity of 95.24%, and a specificity of 87.5% in the training cohort and AUC of 0.981, sensitivity of 94.74%, and specificity of 93.33% in the test cohort. The C-index was 96.80%, and calibration curves showed good agreement between the nomogram predictions and the actual data.

**Conclusions:**

The nomogram showed excellent discrimination and calibration for malignancy risk prediction, and it may aid in making FLLs treatment decisions.

## Introduction

It is challenging for abdominal radiologists to detect and characterize focal liver lesions (FLLs) in patients with cirrhosis (1–3). Cirrhosis is a major risk factor for hepatocellular carcinoma (HCC) (4). The diagnosis of benign and malignant FLLs in cirrhotic individuals is important. However, in cirrhotic livers, these lesions may lack typical imaging features (1). Therefore, the final diagnosis may need to be verified by tissue sampling (1, 5). Accurate descriptions of FLLs guide their management, which ranges from conservative treatment to surgical excision (6). Proper identification can prevent unnecessary biopsy and allow the appropriate treatment to be selected (5, 7).

Computed tomography (CT) is commonly used to diagnose and manage patients with chronic liver disease (8–10). Dynamic CT has high sensitivity and specificity for diagnosing FLLs (11). The wash-in and washout of contrast agents can assist distinguishing HCC from other FLLs (12). Clinical and laboratorial risk factors, such as age and sex, are also helpful. However, many cases require further imaging or histopathological examination to confirm the diagnosis (5, 10). Therefore, it is helpful to develop a scoring system combining clinical information and imaging results to evaluate the malignancy of FLLs.

A nomogram is a graphical statistical tool that combines variables into a continuous scoring system to calculate precise risk probabilities for specific individual outcomes (13–15). This instrument is an important tool in modern medical decision-making in specialties of oncology (16–21), such as differentiating focal nodular hyperplasia (15) or hepatocellular adenoma (16) from HCC in noncirrhotic patients, prediction of microvascular invasion and liver failure after hepatectomy in patients with in HCC (22–25) and efficacy evaluation of intrahepatic cholangiocarcinoma after hepatectomy (26). CT-based nomograms may be used to predict the nature of FLLs, aiding clinicians in selecting the best management plan. We sought to develop a nomogram that incorporates clinical risk factors, blood indicators, and enhanced CT imaging findings to predict the nature of FLLs in cirrhotic livers.

## Material and Methods

### Patients

Our institutional review board approved this retrospective single-center study with a waiver for the requirement to obtain informed consent. The subjects had to have liver cirrhosis of any etiology, surgically confirmed after CT scans between January 2017 and December 2020.

The inclusion criteria were as follows: (a) serological markers, such as serum total bilirubin, plasma albumin, prothrombin time, blood platelet count and α-fetoprotein (AFP), measured simultaneously before surgery; (b) confirmation by pathology; (c) no history of preoperative anticancer therapy, including transcatheter arterial chemoembolization (TACE), percutaneous radiofrequency ablation (PRFA), or percutaneous ethanol injection (PEI); and (d) multidetector CT imaging. The exclusion criteria were as follows: (a) no pathological confirmation; (b) undergoing treatment before imaging or surgery; (c) incomplete serological markers before surgery; and (d) poor image quality. For patients with multiple lesions, we analyzed the largest lesion. A total of 348 surgically confirmed FLLs in 348 patients were included in the study. A total of 295 patients diagnosed from 2017 to June 2020 were included in a training cohort, and 53 patients diagnosed from July 2020 to December 2020 were included in a test cohort.

Age, sex, and basic patient information were collected. Routine examinations included serum total bilirubin, total plasma protein, prothrombin time, blood platelet counts, tumor marker AFP levels, and hepatitis (B and C) results. The Child-Pugh classification was determined for each patient based on the above variables. AFP > 20.0 ng/ml was the threshold for positivity (27).

### CT Technique

The area from the diaphragm to the pubic symphysis was examined on a multidetector CT scanner (Aquilion 16, Toshiba Medical Systems Corporation; Tochigi ken, Japan; Brilliance 64, Philips, Netherlands; Aquilion ONE TSX-301A, Toshiba Medical Systems Corporation; Tochigi ken, Japan) with plain and dynamic contrast-enhanced scans as follows: tube voltage of 120 kVp, tube current of 200 mA, slice thickness of 5 mm, and rotation time of 0.5 seconds. The helical pitch was 0.9, the field of view was 35 to 40 cm, the matrix was 512 × 512, and a standard reconstruction algorithm was used. After plain CT scan, the patients received 80-100 mL of contrast agent (iodipamide, 370 mg I/mL, Bracco) at a rate of 3.5-4.0 mL/s, followed by 20 mL of saline solution through the elbow vein using a power injector. Scans in the arterial phase (AP, 35 seconds), portal venous phase (PVP, 70 seconds), and equilibrium phase (EP, 3 minutes) were obtained.

### CT Imaging Analysis

Two radiologists(7 and 13 years of abdominal diagnostic experience) independently evaluated the CT images from the Picture Archiving and Communication System. They knew the purpose and design of the study, but they were unaware of the patient demographics, clinical history, clinical reports, and reference criteria. For each lesion, the readers assessed the presence of imaging features mainly based on Liver Imaging Reporting and Data System (LI-RADS) version 2018 (28, 29), some features reported in the literature (30, 31) or commonly used in reports. The included features were as follows: tumor size (maximum (Dmax) cross-section diameter), non-rim arterial phase hyperenhancement (APHE), non-peripheral washout in the PVP (washout), enhancing capsule in either the portal venous or delay phases (appearance detected as enhancing rim), blood product in mass (bleeding within or around the lesion without surgery, trauma or intervention), fat (excess fat in the whole or part of the mass relative to the background liver), necrosis (areas within the tumor without obvious enhancement), infiltrative appearance (invasion), mural nodules (peripheral nodules within the lesion attached to the tumor wall), satellite lesions (nodules in the surrounding parenchyma resemble the main lesion), halo enhancement(solar enhancement in parenchyma around the lesion), peritumoral enhance(rim arterial phase hyperenhancement), vein tumor thrombus (VTT, definite enhancement of soft tissue in the portal vein), delayed enhancement(progressive enhancement in the center of the lesion), internal artery (small vessels in the arterial stage), non-enhancing “capsule” (capsule appearance not detected as enhancing rim), mosaic architecture(random distribution of internal nodules or compartments, often with different radiographic features), nodule-in-nodule architecture (the internal nodules were small and larger than the external nodules, with different imaging features), corona enhancement, lymph node enlargement (short diameter >10mm) and ascites (perihepatic water density).

### Statistical Analysis

Statistical analysis was performed using IBM SPSS Statistics version 25 (SPSS Inc., Chicago, IL, USA) and R version 3.3.4 (www.R-project.org). The Mann-Whitney U test was used for continuous variables, and the *χ* ^2^ or Fisher exact test was used for categorical variables. To test the consistency of the two readers, a Kendall correlation coefficient was used to measure the index, and a kappa consistency test was used for the counting index. The k values were considered poor for a k of 0.01 to 0.20; fair for a k of 0.21 to 0.40; moderate for a k of 0.41 to 0.60; good for a k of 0.61 to 0.80; and excellent for a k of 0.81 to 1.00. Univariate analysis was used to compare the differences in clinical factors (all independent clinical risk factors, blood markers, and CT findings) between the two cohorts, and multivariate logistic regression analysis was used to establish a clinical factor model, with significant variables in the univariate analysis as the input. The odds ratio (OR) was used as a relative risk estimate for each risk factor and is presented with its corresponding 95% confidence interval (CI). After establishing the combined predictor, receiver operating characteristic (ROC) analysis was performed to calculate the area under the curve (AUC), sensitivity and specificity. A nomogram was developed by scaling the regression coefficients into a multiple logistic regression of 0-100 points. Important malignancy factors in the multivariate analysis were included in the nomograms. The total score is the sum of the points for each independent variable and is converted to the prediction probability. The nomogram’s performance was measured by the consistency index (C-index) and calibrated with 1,000 bootstrap samples to reduce overfitting bias (32). A calibration curve was plotted to evaluate the actual observations *vs* the nomogram predictions of the benignity or malignancy of lesions. Decision curve analysis (DCA) showed that the net clinical benefit was correlated with the diagnostic procedure including the established nomogram (33). All statistical tests were two-sided, and a P-value < 0.05 was considered statistically significant.

## Results

### Histopathologic Results

**Figure 1:**
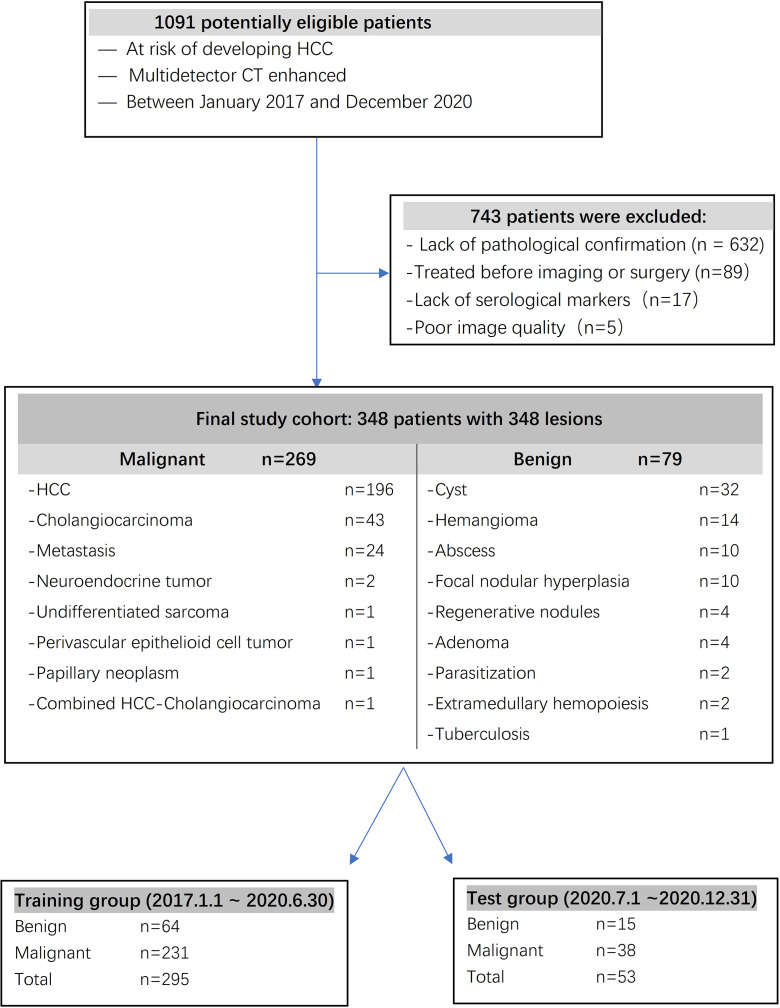
Flowchart illustrating subject selection.

A total of 348 FLLs were identified in the patients, including HCC (n = 196), cholangiocarcinoma (n = 43), metastasis (n = 24), neuroendocrine tumor (n = 2), undifferentiated sarcoma (n = 1), perivascular epithelioid cell tumor (n = 1), papillary neoplasm (n = 1), combined HCC- cholangiocarcinoma (n = 1), cyst (n = 32), hemangioma (n = 14), abscess (n = 10), focal nodular hyperplasia (n = 10), regenerative nodules (n = 4), adenoma (n = 4), parasitization (n = 2), extramedullary hemopoiesis (n = 2), and tuberculosis (n = 1). These FLLs were either benign (n = 79) or malignant (n = 269), as shown in **Figure 1**, and the baseline characteristics of the patients are shown in **Table 1** and **Table 2**.

**Table 1:** Characteristics of patients in the training and test cohorts (1).

Parameters		Training (n = 295)					Test (n = 53)				
		Benign	Malignant	Total	χ^2^	P	Benign	Malignant	Total	χ^2^	P
**Sex**	Male	34	186	220	19.836	<0.001	11	33	44	1.39	0.238
	Female	30	45	75			4	5	9		
**CGOLF**	A	52	156	208	4.764	0.092	12	29	41	0.08	0.773
	B	12	73	85			3	9	12		
	C	0	2	2			0	0	0		
**Pathogenesis**	Alcohol	39	29	68	66.657	<0.001	10	5	15	15.18	<0.001
	HBV	25	195	220			5	33	38		
	HCV	0	6	6			0	0	0		
	PBC	0	1	1			0	0	0		
**AFP**	-	60	89	149	61.136	<0.001	15	14	29	17.31	<0.001
	+	4	142	146			0	24	24		
**APHE**	-	59	63	122	87.075	<0.001	14	7	21	25.23	<0.001
	+	5	168	173			1	31	32		
**Washout**	-	50	96	146	26.807	<0.001	12	15	27	7.07	0.008
	+	14	135	149			3	23	26		
**Capsule**	-	30	71	101	5.798	0.016	9	11	20	4.41	0.036
	+	34	160	194			6	27	33		
**Blood**	-	56	156	212	9.882	0.002	13	22	35	3.97	0.046
	+	8	75	83			2	16	18		
**Necrosis**	-	26	28	54	27.228	<0.001	4	3	7	3.31	0.069
	+	38	203	241			11	35	46		
**Infiltrative**	-	56	157	213	9.529	0.002	13	24	37	2.82	0.093
	+	8	74	82			2	14	16		
**Mural**	-	31	74	105	5.882	0.015	6	13	19	0.16	0.692
	+	33	157	190			9	25	34		
**Satellite**	-	56	157	213	9.529	0.002	13	23	36	3.37	0.066
	+	8	74	82			2	15	17		
**Halo**	-	58	201	259	0.610	0.435	14	32	46	0.78	0.377
	+	6	30	36			1	6	7		
**PE**	-	51	183	234	0.007	0.935	12	33	45	0.39	0.531
	+	13	48	61			3	5	8		
**VTT**	-	64	210	274	6.264	0.012	15	34	49	1.71	0.191
	+	0	21	21			0	4	4		
**DE**	-	58	213	271	0.168	0.682	13	36	49	1.00	0.316
	+	6	18	24			2	2	4		
**Internal**	-	42	36	78	64.521	<0.001	10	9	19	8.64	0.003
	+	22	195	217			5	29	34		
**LN**	-	63	220	283	1.315	0.252	15	35	50	1.26	0.263
	+	1	11	12			0	3	3		
**Ascites**	-	63	205	268	5.663	0.017	15	35	50	1.26	0.263
	+	1	26	27			0	3	3		
**Fat**	-	63	227	290	0.009	0.926	–	–	–	–	–
	+	1	4	5			–	–	–		
**NC**	-	34	207	241	44.611	<0.001	11	36	47	4.91	0.027
	+	30	24	54			4	2	6		
**Mosaic**	-	35	26	61	57.635	<0.001	9	8	17	7.49	0.006
	+	29	205	234			6	30	36		
**Nodule**	-	58	81	139	62.087	<0.001	15	17	32	13.73	<0.001
	+	6	150	156			0	21	21		
**Corona**	-	64	221	285	2.868	0.090	15	36	51	0.82	0.365
	+	0	10	10			0	2	2		

CGOLF, Child grading of liver function; HBV, Hepatitis C virus; HCV, Hepatitis C virus; PBC, Primary biliary cirrhosis; APHE, non-rim Arterial phase hyperenhancement; Blood, Blood product in mass; Infiltrative, infiltrative appearance; Mural, Mural nodules; Satellite, Satellite lesions; Halo, Halo enhancement; PE, Peritumoral enhancement; VTT, vein tumor thrombus; DE, Delayed enhancement; Internal, Internal artery; LN, Lymph node; NC, Non-enhancing “capsule”; Mosaic, Mosaic architecture; Nodule, Nodule-in-nodule architecture; Corona, Corona enhancement.

**Table 2:** Characteristics of patients in the training and test cohorts (2).

Parameters	Training								Test							
	Benign			Malignant			Z	P	Benign			Malignant			Z	P
	25th	Median	75th	25th	Median	75th			25th	Median	75th	25th	Median	75th		
**Age(year)**	49.3	56.0	64.0	47.0	56.0	63.0	-0.697	0.486	46.0	58.0	65.0	50.5	57.0	63.0	-0.761	0.446
**STB (umol/L)**	12.1	21.4	43.4	15.4	25.0	59.5	2.029	0.042	12.4	23.1	69.4	13.7	20.8	34.8	-0.227	0.820
**PA(g/L)**	14.4	63.9	73.9	18.9	63.0	68.4	-0.419	0.675	11.2	19.0	71.8	21.8	65.0	74.0	1.343	0.179
**PT(s)**	12.6	13.6	14.3	12.9	13.7	14.3	1.260	0.208	13.0	13.9	14.6	12.8	14.1	15.9	0.445	0.657
**BP (X10^9^/L)**	173.5	234.5	289.3	138.0	189.0	251.0	-3.073	0.002	173.0	206.0	284.0	158.8	187.5	289.0	-0.632	0.527
**Dmax (cm)**	4.8	6.5	8.7	3.7	5.4	8.7	-1.848	0.065	4.4	6.5	8.0	3.4	4.9	8.8	-0.622	0.534

STB, Serum total bilirubin; PA, Plasma albumin; BP, Blood platelet; PT, Prothrombin time; Dmax, Maximum cross-section diameter.

### Interobserver Agreement

The indexes of lesion size (Dmax) had good consistency among observers (P > 0.05). The consistency value of the counting indexes among observers was greater than 0.75, indicating good consistency between observers.

### Univariate Analysis of Independent Predictors of Malignancy

For categorical variables, sex (*χ* ^2^ = 19.836, P < 0.001), pathogenesis (*χ* ^2^ = 66.657, P < 0.001), AFP (*χ* ^2^ = 61.136, P < 0.001), APHE (*χ* ^2^ = 87.075, P < 0.001), washout (*χ* ^2^ = 26.807, P < 0.001), enhancing capsule (*χ* ^2^ = 5.798, P = 0.016), blood product in mass (*χ*2 = 9.882, P = 0.002), necrosis (*χ* ^2^ = 27.228, P < 0.001), infiltrative appearance (*χ* ^2^ = 9.529, P=0.002), mural nodules (*χ* ^2^ = 5.882, P = 0.015), satellite lesions (*χ* ^2^ = 9.529, P = 0.002), VTT (*χ* ^2^ = 6.264, P = 0.012), internal artery (*χ* ^2^ = 64.521, P < 0.001), ascites (*χ* ^2^ = 5.663, P=0.017), non-enhancing capsule (*χ* ^2^ = 44.611, P < 0.001), mosaic architecture (*χ* ^2^ = 57.635, P < 0.001), and nodule-in-nodule architecture (*χ* ^2^ = 62.087, P < 0.001) were significantly different between the two cohorts (**Table 1**). There were significant differences in blood platelet counts (P =0.002) and serum total bilirubin (P =0.042) between the benign and malignant cohorts, but there were no significant differences in the other indexes (P > 0.05) (**Table 2**).

### Multivariable Factors Associated With Malignancy

**Figure 2:**
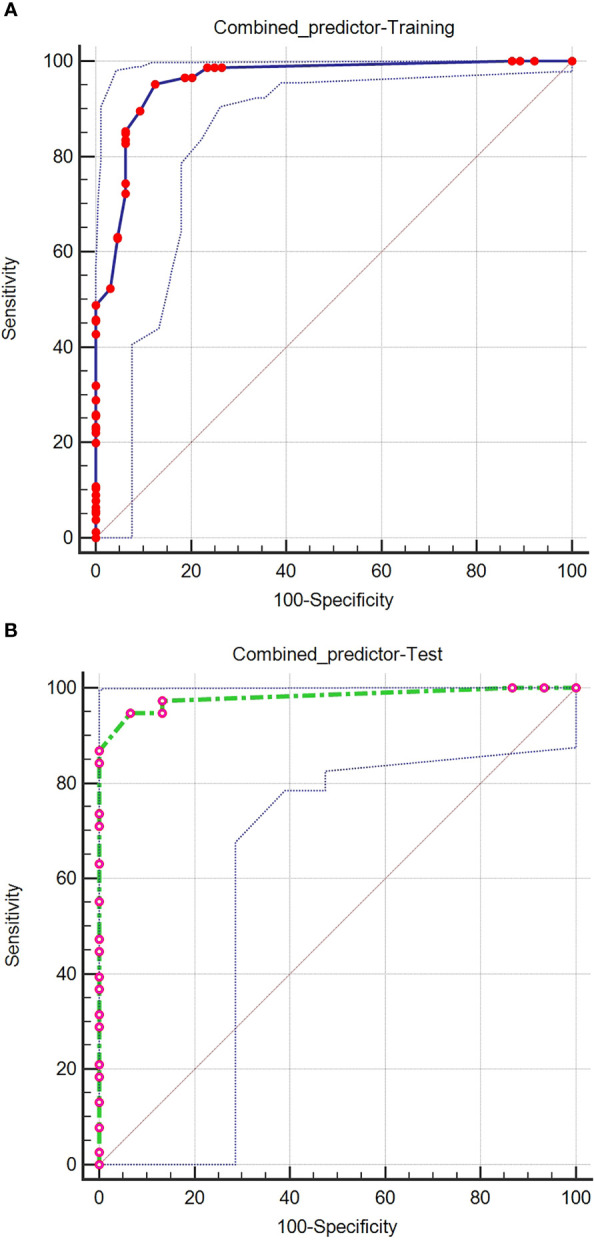
ROC curves of the combined predictors for predicting malignant lesions in the training **(A)** and test **(B)** cohorts.

Logistic regression evaluated the independent factors affecting malignancy. AFP (OR = 10.547, 95% CI 2.083- 53.401; P = 0.004), APHE (OR = 740.876, 95% CI 56.527- 9710.303; P < 0.001), washout (OR = 0.028, 95% CI 0.002-0.348; P = 0.005), satellite lesions (OR = 15.164, 95% CI 2.199-104.579; P = 0.006), ascites (OR = 156.241, 95% CI 1.822-13394.835; P = 0.026), and nodule-in-nodule architecture (OR = 27.401, 95% CI 4.982-150.700; P < 0.001) were independent predictors of malignancy (**Table 3**). The independent factors are used to build a model as combined predictors. The results of ROC analysis of the combined predictors for predicting malignant lesions are shown in (**Figures 2A, B**). In the training cohort, the AUC was 0.959, the sensitivity was 95.24%, and the specificity was 87.50%, while in the test cohort, the AUC was 0.981, the sensitivity was 94.74%, and the specificity was 93.33%. The order of AUCs was as follows: test cohort > training cohort > APHE > nodule-in-nodule architecture > AFP > washout > satellite lesions > ascites (**Table 4**).

**Table 3:** The results of multivariate logistic regression analysis.

Parameters	B	S.E.	Wald	P	OR	95% C.I. for OR	
						Lower	Upper
**Sex**	-0.477	0.673	0.503	0.478	0.621	0.166	2.320
**Pathogenesis**	–	–	0.402	0.940	–	–	–
**Pathogenesis (1**)	-14.731	40193.1	0.000	1.000	0	0	–
**Pathogenesis (2**)	-14.257	40193.1	0.000	1.000	0	0	–
**Pathogenesis (3**)	1.931	42542.7	0.000	1.000	6.895	0	–
**AFP**	2.356	0.828	8.104	0.004	10.547	2.083	53.401
**APHE**	6.608	1.313	25.334	0.000	740.876	56.527	9710.303
**Washout**	-3.564	1.279	7.762	0.005	0.028	0.002	0.348
**Capsule**	0.993	0.726	1.867	0.172	2.698	0.650	11.207
**Blood**	1.451	0.962	2.272	0.132	4.266	0.647	28.140
**Necrosis**	-1.186	0.926	1.641	0.200	0.306	0.050	1.875
**Infiltrative**	-0.002	0.888	0.000	0.998	0.998	0.175	5.688
**Mural nodules**	-0.445	0.721	0.381	0.537	0.641	0.156	2.632
**Satellite lesions**	2.719	0.985	7.616	0.006	15.164	2.199	104.579
**DE**	18.150	6298.88	0.000	0.998	7.6E+07	0	–
**Internal**	0.797	0.884	0.813	0.367	2.219	0.392	12.555
**Ascites**	5.051	2.271	4.947	0.026	156.241	1.822	13394.835
**NC**	-1.410	1.033	1.863	0.172	0.244	0.032	1.849
**Mosaic**	0.035	0.945	0.001	0.971	1.035	0.162	6.596
**Nodule**	3.311	0.870	14.488	0.000	27.401	4.982	150.700
**STB**	0.021	0.014	2.161	0.142	1.021	0.993	1.050
**Blood platelet**	0.001	0.003	0.243	0.622	1.001	0.996	1.007
**Constant**	11.839	40193.1	0.000	1.000	1	138612.5	–

APHE, non-rim Arterial phase hyperenhancement; Blood, Blood products in mass; DE, Delayed enhancement; Internal, Internal artery; NC, Non-enhancing “capsule”; Mosaic, Mosaic architecture; Nodule, Nodule-in-nodule architecture; STB, Serum total bilirubin.

**Table 4:** Results of receiver operating characteristic (ROC) analysis.

Group	AUC	95%CI	Youden index	Sensitivity(%)	Specificity(%)	P
**Training#**	0.959	0.929,0.978	0.827	95.24	87.50	<0.001
**Test#**	0.981	0.899,0.999	0.881	94.74	93.33	<0.001
**AFP**	0.776	0.724,0.822	0.552	61.47	93.75	<0.001
**APHE**	0.825	0.776,0.866	0.649	72.73	92.19	<0.001
**Washout**	0.683	0.626,0.736	0.366	58.44	78.12	<0.001
**Satellite lesions**	0.598	0.539,0.654	0.195	32.03	87.50	<0.001
**Ascites**	0.548	0.490,0.606	0.097	11.26	98.44	<0.001
**Nodule**	0.778	0.726,0.824	0.556	64.94	90.62	<0.001

# as combined predictors.

APHE, non-rim Arterial phase hyperenhancement; Nodule, Nodule-in-nodule architecture.

### Malignancy Risk and the Prediction Nomogram

**Figure 3:**
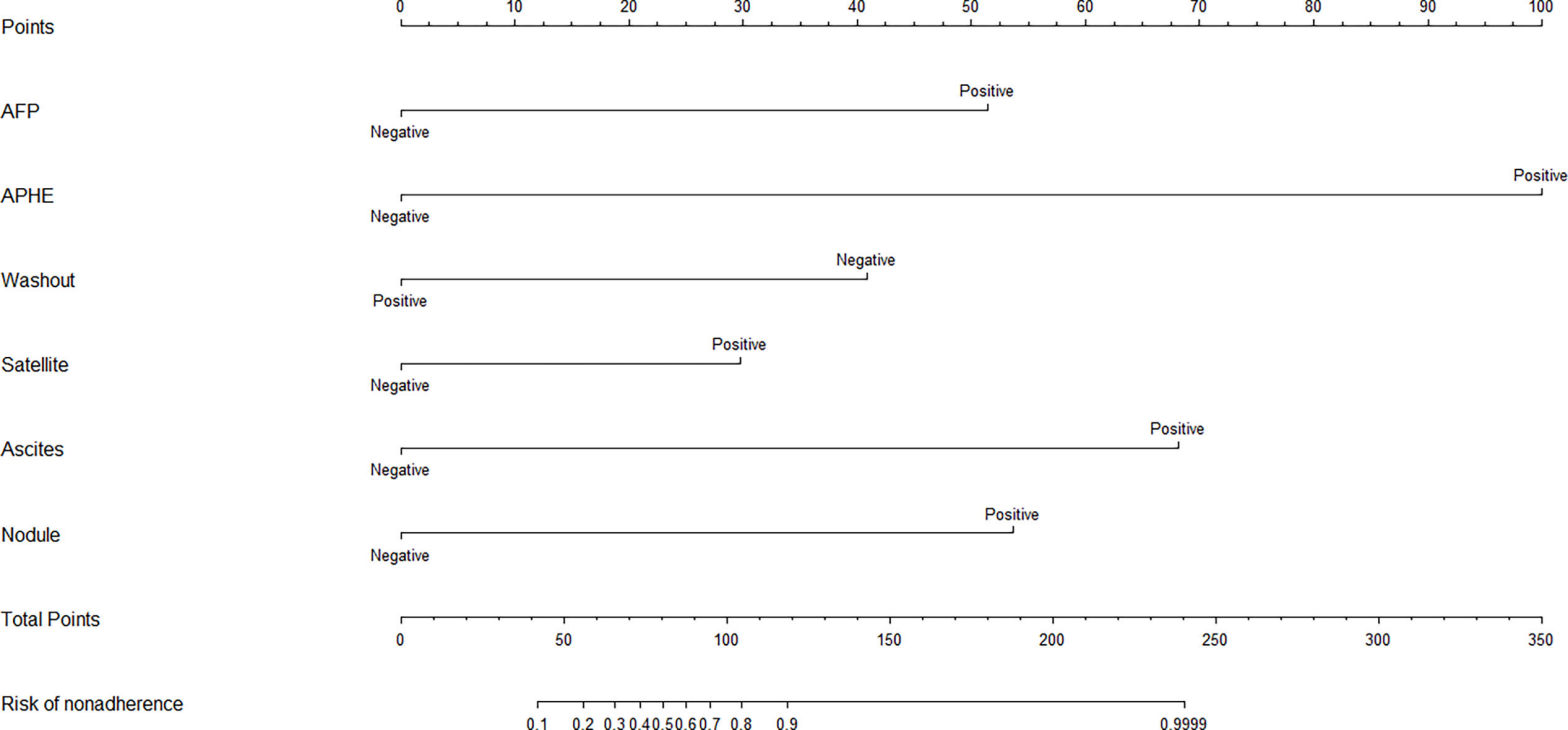
Based on the independent factors, the nomogram analysis method was used to establish a prediction scoring system. To use the nomogram, find the score for each variable on the corresponding axis and the total scores for all of the variables, and draw a line from the total score axis to the malignant risk axis to determine the malignancy risk (APHE, nonrim Arterial phase hyperenhancement; Satellite,Satellite lesions; Nodule,Nodule-in-nodule architecture).

**Figure 4:**
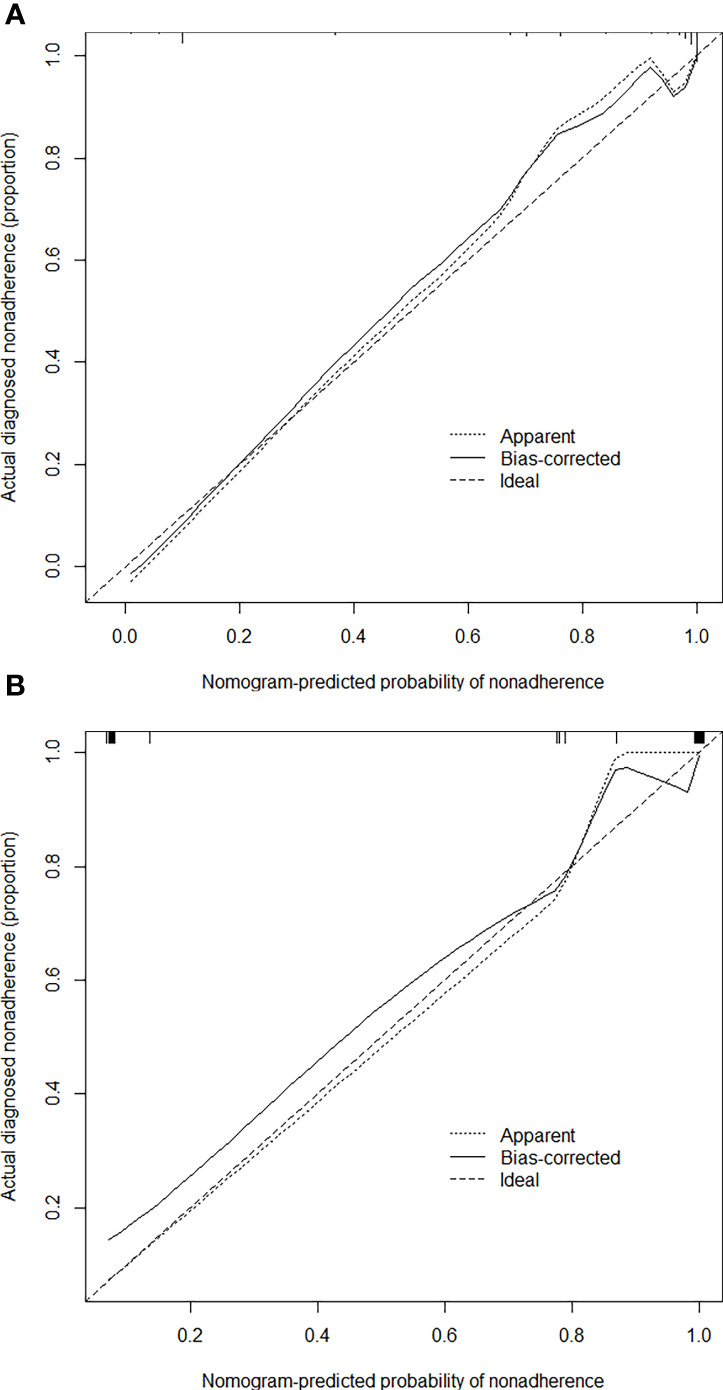
Calibration curves of the nomogram for estimating the malignancy risk in the training **(A)** and test **(B)** cohorts. On the calibration curve, the x-axis is the nomogram-predicted probability of malignancy, and the y-axis is the actual probability. The dotted line represents the ideal curve; the small, dotted line is the nomogram curve; and the straight line is the bias-corrected curve.

**Figure 5:**
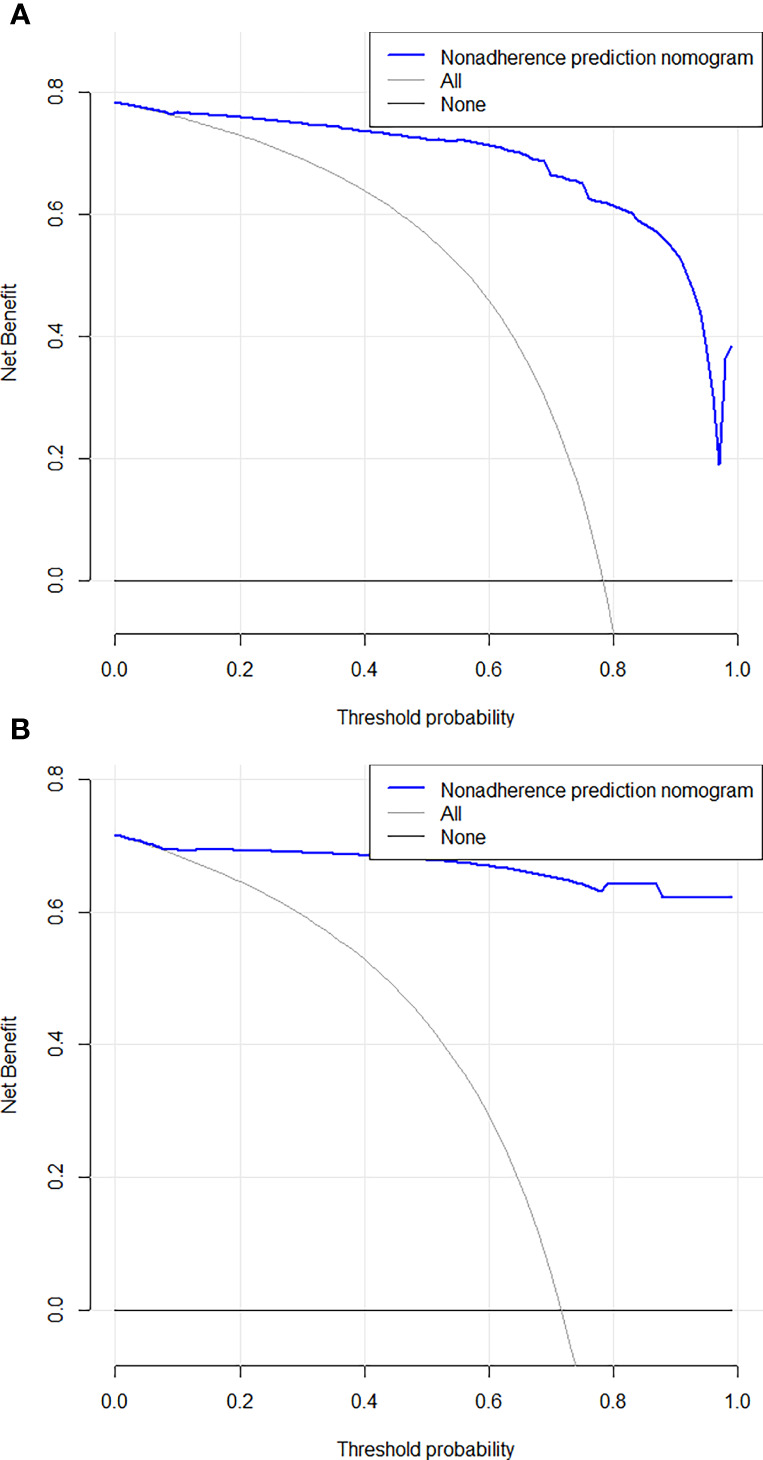
Decision curve analysis for the combined nomogram in the training **(A)** and test **(B)** cohorts. The Y axis represents the net benefit, and the X axis represents the threshold probability. The solid blue line shows the expected net benefit per patient based on Nomogram.

Based on the independent factors, we established a nomogram of the corresponding scoring system using RMS package in R as shown in **Figure 3**. Total points = 51 (AFP) + 100 (APHE) **−** 41 (washout) + 30 (satellite lesions) + 68 (ascites) + 54 (nodule-in-nodule architecture). The probability of malignant FLLs was approximately 80% for a patient with a score of 104, and with a cutoff point of 50%, the lesion was considered malignant. With a 50% cutoff point, a score of more than 80 points indicated malignant FLLs with a C-index of 96.80%. Moreover, calibration curves showed good agreement between the nomogram predictions and the actual data (**Figures 4A, B**). The DCA results are shown in **Figures 5A, B**.

## Discussion

In this study, we established a precise nomogram based on CT imaging findings for predicting the malignancy of FLLs. The results indicate a good identification effect.

Triphasic CT scans are effective tools for differentiating benign and malignant FLLs, as the sensitivity, specificity, positive predictive value, negative predictive value, and diagnostic accuracy were 100%, 80%, 94.5%, 100% and 95.5%, respectively (34). Diffusion weighted imaging (DWI) may provide additional information for the differentiation of HCC from nodules with abnormal hyperplasia by detecting the movement of freely diffusible water molecules. DWI model has a reference value for describing FLLs, the distributed diffusion coefficient, which shows good diagnostic performance (35).

In the univariate analysis of independent predictors of malignancy, most factors showed significant differences between the benign and malignant cohorts. Male patients with higher serum total bilirubin, higher AFP levels and lower blood platelet counts may be prone to malignant lesions. In CT, the presence of the following features indicates a high possibility of malignant lesions: APHE, washout, enhancing capsule, blood product in mass, necrosis, infiltrative appearance, mural nodules, satellite lesions, VTT, internal artery, ascites, non-enhancing capsule, mosaic architecture, and nodule-in-nodule architecture. Multivariable factors including AFP, APHE, washout, satellite lesions, ascites, and nodule-in-nodule architecture, were independent predictors of malignancy. A previous study reported that capsular enhancement is an important imaging biomarker for predicting high-grade HCC, and non-enhancing capsule is not significantly associated with high-grade HCC (36). However, our study showed that in our cohort, capsular enhancement was not an independent predictor of malignancy. The reason may be that some of the benign lesions such as abscesses, also showed capsular enhancement. APHE and washout are the main features of HCC, while nodule-in-nodule architecture is an auxiliary feature of HCC (37, 38). Nodule-in-nodule architecture, defined as a small nodule within the lesion, is an independent predictor of microvascular invasion (MVI) of HCC (36) and is proven to be an independent predictor of malignancy in our study. HCC accounts for the majority of malignant lesions, and these three features are independent predictors for the identification of benign and malignant FLLs in this study. A diagnostic model including AFP, sex, age and prothrombin time (ASAP model) has been shown to accurately predict the development of HCC in patients at high risk of hepatitis B virus. The ASAP model performed well in both the test and validation groups (39). In our study, with the exception of AFP, sex, serum total bilirubin, blood platelet counts, and pathogenesis were not independent predictors of malignancy, although there were statistically significant differences in these factors between the benign and malignant cohorts. The order of AUC values suggested that the combined predictors were the strongest in the diagnosis of malignant lesions (sensitivity = 95.24%, specificity = 87.50%). This model also had high diagnostic sensitivity (94.74%) and specificity (93.33%) in the test group.

**Figure 6:**
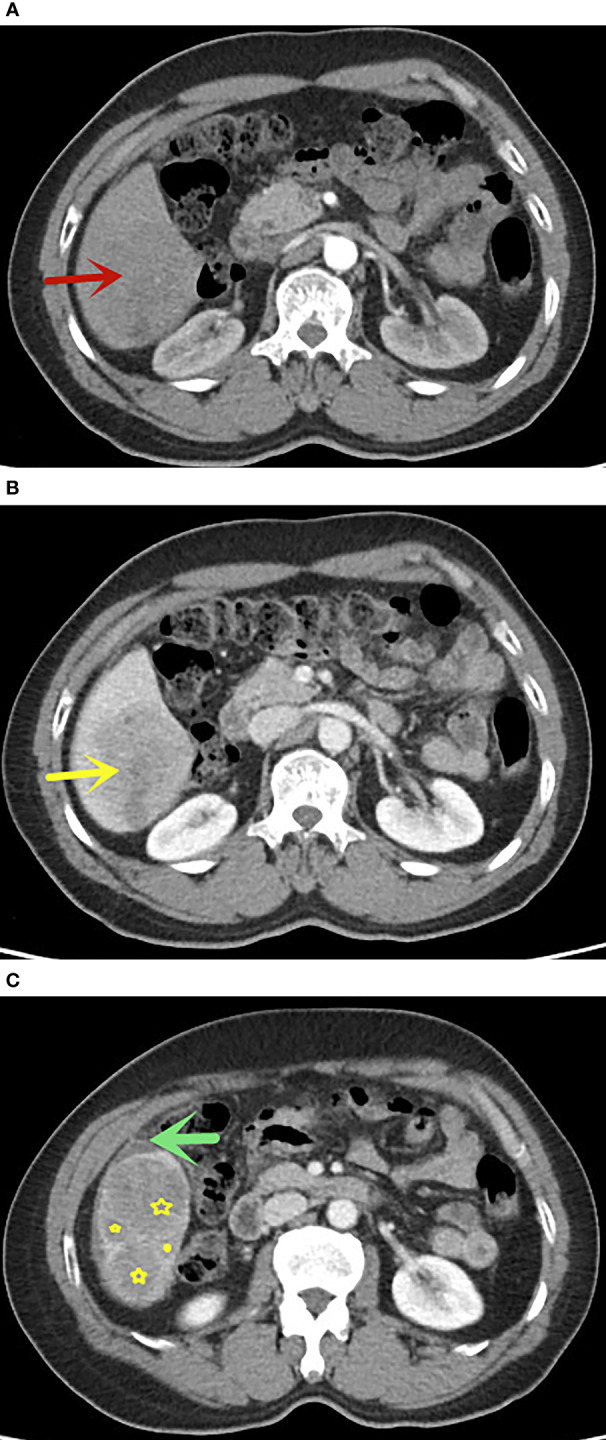
A 58 years old man with FLLs in segment VI, AFP>20.0 ng/ml, **(A)** no APHE in arterial phase (red arrow), **(B)** washout in portal venous phase, **(C)** presence of nodule-in-nodule architecture(yellow star) and ascites(green arrow), without satellite lesions. This lesion indicates malignancy according to the nomogram score greater than 80 points. The pathological result was hepatocellular carcinoma.

We established a nomogram with a corresponding scoring system. With a cutoff point of 50%, it can accurately determine if an FLL is malignant. An example of image scoring is illustrated (**Figures 6A–C**).This lesion demonstrates no arterial phase hyperenhancement (APHE,0 score points, **Figure 6A**), presence of washout (minus 41 score points, **Figure 6B**), without satellite lesions (0 score points), ascites (68 score points, **Figure 6C**) and nodule-in-nodule architecture (54 score points, **Figure 6C**), with AFP>20.0 ng/ml (51 score points). The nomogram equation therefore would be as follows: 51 (AFP) + 100 (APHE) -41 (washout) + 30 (satellite lesions) + 68 (ascites) + 54 (nodule-in-nodule architecture) =132, indication malignancy according to the nomogram score > 80 points. Washout is one of the main features in the diagnosis of HCC, but in our study, it was negatively associated with benign and malignant tumors. One of the possible reasons is that some benign lesions, such as adenomas, can show washout, while some HCC lesions lack washout. In addition, washout is a purely visual criterion, which may result in observer-dependent bias. According to the literature reports, quantitative washout assessment in LI-RADS has the opposite effect in HCC diagnosis and needs to be redefined (40).

In this study, the C-index (96.80%) and calibration curve demonstrated that our nomogram was accurate in predicting the malignancy of lesions. However, there are some limitations to our model. First, this was a small, single-center, retrospective study that lacked a external validation group, which may alter the scoring system’s efficiency. Therefore, multicenter and large-scale studies are necessary to improve the scoring system, and a prospective study is needed to confirm its reliability. Second, cirrhosis is likely to increase the possibility of HCC, which may cause selective bias. Third, rare lesions of liver were really small in our study. Assessing more data can make the model more generalizable.

## Conclusion

Based on AFP and CT findings including APHE, washout, satellite lesions, ascites, and nodule-in-nodule architecture, we developed an objective scoring system to predict the risk of malignancy. This model may aid in making informed treatment decisions for FLLs. A large-scale, prospective validation study is needed to assess the broad applicability of the nomogram.

## Data Availability Statement

The original contributions presented in the study are included in the article/supplementary material. Further inquiries can be directed to the corresponding author.

## Ethics Statement

This retrospective study was approved by the institutional review board of the hospital (K-2019-079-02) and based on the principles of the Helsinki declaration.

## Author Contributions

HW, ZW, and CT designed the study, analyzed the data and drafted manuscript. XW, WZ, LM, and XJ contributed significantly to revise the manuscript. YL and RY polished the language. All authors contributed to the article and approved the submitted version.

## Funding

This work has received funding from the Science Foundation of Guangzhou First People’s Hospital (M2019013), Guangzhou Health Science and Technology Project (20201A011013, 20211A011013), the Traditional Chinese Medicine Scientific Research Project of Guangdong Traditional Chinese Medicine Bureau (20201290), the Science and Technology Project of Guangzhou (201904010422, 202102010025), the Natural Science Foundation of Guangdong Province (2021A1515011350) and the Special Fund for the Construction of High-level Key Clinical Specialty (Medical Imaging) in Guangzhou Guangzhou Key Laboratory of Molecular Imaging and Clinical Translational Medicine.

## Conflict of Interest

The authors declare that the research was conducted in the absence of any commercial or financial relationships that could be construed as a potential conflict of interest.

## Publisher’s Note

All claims expressed in this article are solely those of the authors and do not necessarily represent those of their affiliated organizations, or those of the publisher, the editors and the reviewers. Any product that may be evaluated in this article, or claim that may be made by its manufacturer, is not guaranteed or endorsed by the publisher.
